# Unilateral branch retinal vein occlusion and contralateral branch retinal artery occlusion: a case report and comprehensive literature review

**DOI:** 10.3389/fmed.2025.1615871

**Published:** 2025-07-22

**Authors:** Chao Zhang, Han Wang, Zhiyu Xu, Qiwei Lu, Ying Zhu, Jun Li

**Affiliations:** ^1^Department of Ophthalmology, The Third People's Hospital of Dalian, Dalian, Liaoning, China; ^2^Department of Retina, He Eye Specialists Hospital of Shenyang, Shenyang, China

**Keywords:** branch retinal vein occlusion, branch retinal artery occlusion, retinal ischemia, vascular risk factors, diabetes, hypertension

## Abstract

Branch retinal vein occlusion (BRVO) and branch retinal artery occlusion (BRAO) are two distinct yet serious retinal vascular disorders that often present with acute visual impairment. While both conditions are commonly encountered independently, their simultaneous or sequential occurrence in opposite eyes of the same patient is exceedingly rare and poses unique diagnostic and therapeutic challenges. Here, we present the case of a 65-year-old male patient with a history of systemic hypertension who developed BRAO in the right eye and BRVO in the left eye within a short time interval. Detailed ophthalmologic examination, multimodal imaging including optical coherence tomography (OCT) and fundus fluorescein angiography (FFA), and systemic vascular assessments such as carotid Doppler ultrasonography and cardiac evaluation were performed to identify the underlying etiology and guide treatment decisions. The patient was found to have significant carotid atherosclerosis with plaque formation, suggesting systemic vascular pathology as a shared risk factor for both events. Ocular treatment involved posterior sub-Tenon injection of corticosteroids, vasodilator therapy, and laser photocoagulation for ischemic areas. Visual acuity showed marked improvement in both eyes after appropriate interventions and systemic blood pressure control. The concurrent presentation of BRVO and BRAO in different eyes may reflect a systemic vascular disease burden and warrants a high index of suspicion for underlying cardiovascular or cerebrovascular abnormalities. This case underscores the importance of prompt ocular diagnosis, thorough systemic evaluation, and multidisciplinary collaboration to prevent further vascular events and preserve visual function. A detailed literature review is also provided to contextualize the clinical features, diagnostic tools, management approaches, and prognosis of such rare bilateral retinal vascular presentations.

## Introduction

Retinal vascular occlusions are among the leading causes of sudden, painless visual loss, particularly in middle-aged and elderly populations. These occlusive events are broadly classified into arterial and venous types, with branch retinal vein occlusion (BRVO) and branch retinal artery occlusion (BRAO) representing the most common subtypes of each category, respectively (1). BRVO occurs when one of the smaller branches of the central retinal vein becomes obstructed, typically at arteriovenous crossings where a thickened, atherosclerotic artery compresses the underlying vein. This leads to increased venous pressure, retinal hemorrhages, venous dilation, macular edema, and consequent vision impairment (2, 3). BRAO, in contrast, arises from obstruction of a retinal arterial branch, usually due to emboli originating from carotid artery atherosclerosis or cardiac sources, resulting in acute retinal ischemia, retinal whitening along the distribution of the affected artery, and a classic “cherry-red spot” at the macula if the fovea remains perfused by the underlying choroid (4, 5).

Although both BRVO and BRAO are relatively common when considered separately, their simultaneous or sequential occurrence in different eyes of the same patient is exceptionally rare and only sporadically reported in the literature. The pathophysiological mechanisms underlying these two conditions are distinct—venous occlusion is primarily related to mechanical compression and thrombosis, whereas arterial occlusion is usually embolic or vasospastic in nature (6–8). However, they share several systemic vascular risk factors, most notably systemic hypertension, diabetes mellitus, hyperlipidemia, smoking, and advanced age (3). Additionally, conditions that promote atherosclerosis and vascular endothelial dysfunction may predispose a patient to both types of occlusions, albeit through different pathways.

Importantly, the presence of both BRVO and BRAO in the same patient, though affecting opposite eyes, may reflect an advanced state of systemic vascular disease and herald an increased risk of cerebrovascular or cardiovascular events. Therefore, such presentations should prompt a thorough systemic evaluation, including cardiovascular and cerebrovascular risk assessment, carotid artery imaging, and cardiac workup to identify potential embolic sources or vascular compromise. Ocular imaging modalities such as fundus fluorescein angiography (FFA) and optical coherence tomography (OCT) are invaluable in confirming the diagnosis, assessing perfusion status, and guiding treatment strategies.

In this report, we present a rare case of branch retinal artery occlusion in one eye and branch retinal vein occlusion in the fellow eye in a 65-year-old male patient with a background of systemic hypertension and recent cataract surgery. We also provide a comprehensive review of the existing literature to summarize the clinical features, diagnostic modalities, pathophysiological mechanisms, management strategies, and long-term visual prognosis associated with these uncommon but vision-threatening conditions. This case serves to highlight the importance of recognizing ocular vascular events as potential markers of systemic vascular pathology and the value of a multidisciplinary approach to both ocular and systemic disease management. This study was approved by the Ethics Committee of The Third People’s Hospital of Dalian (Approval No. 2025-080-001).

## Case presentation

**Figure 1:**
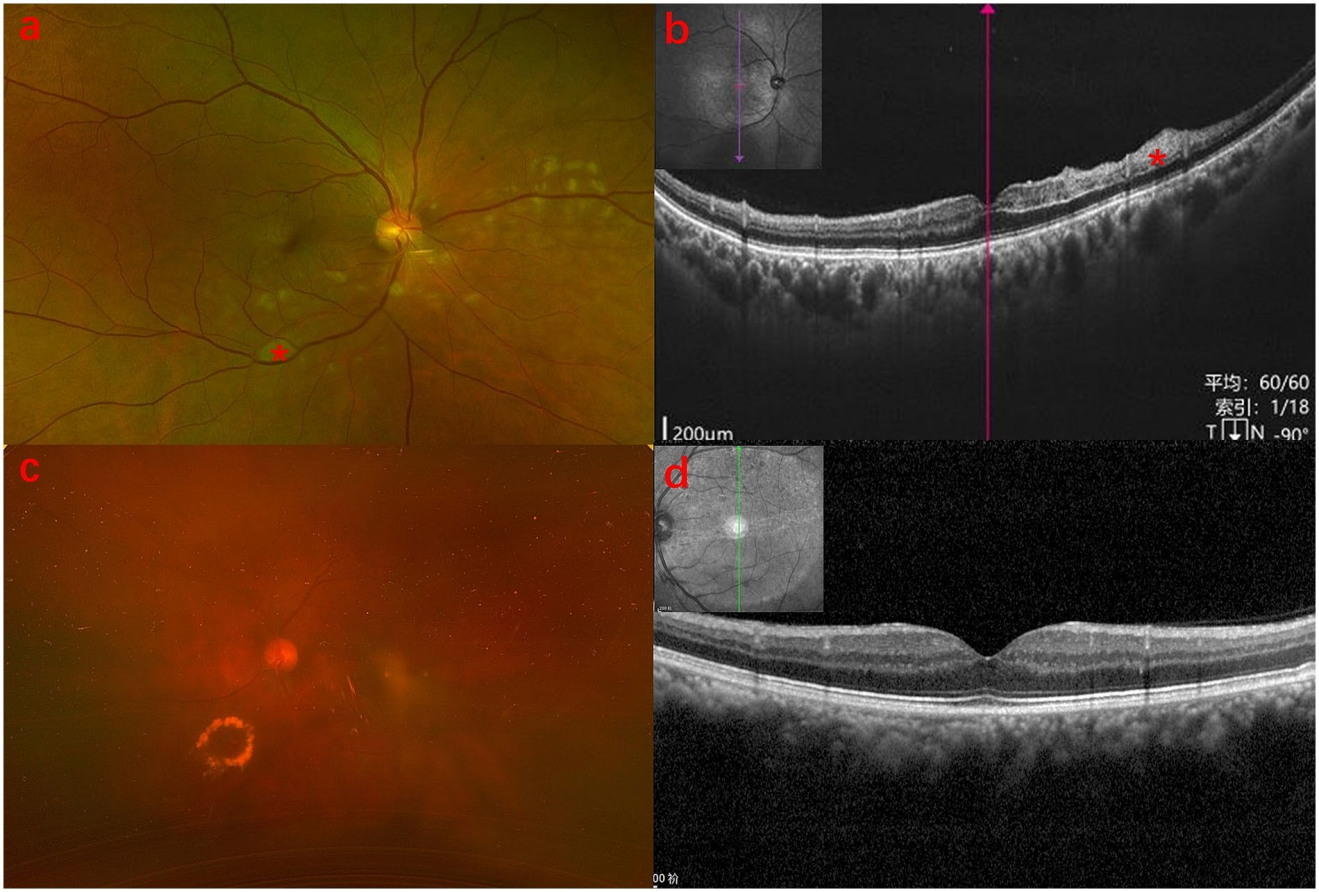
Pre-treatment fundus photography and OCT findings. **(a)** Fundus photo (BRAO eye): multiple cotton-wool spots and retinal pallor with edema in the posterior pole. **(b)** OCT (BRAO eye): significant macular edema with increased reflectivity and thickening of the inner retina; * Indicates the area corresponding to show retinal whitening and poor layer differentiation. **(c)** Fundus photo (BRVO eye): retinal details are obscured by cataract; ring-shaped exudates observed inferonasally. **(d)** OCT (BRVO eye): no significant retinal edema detected.

**Figure 2:**
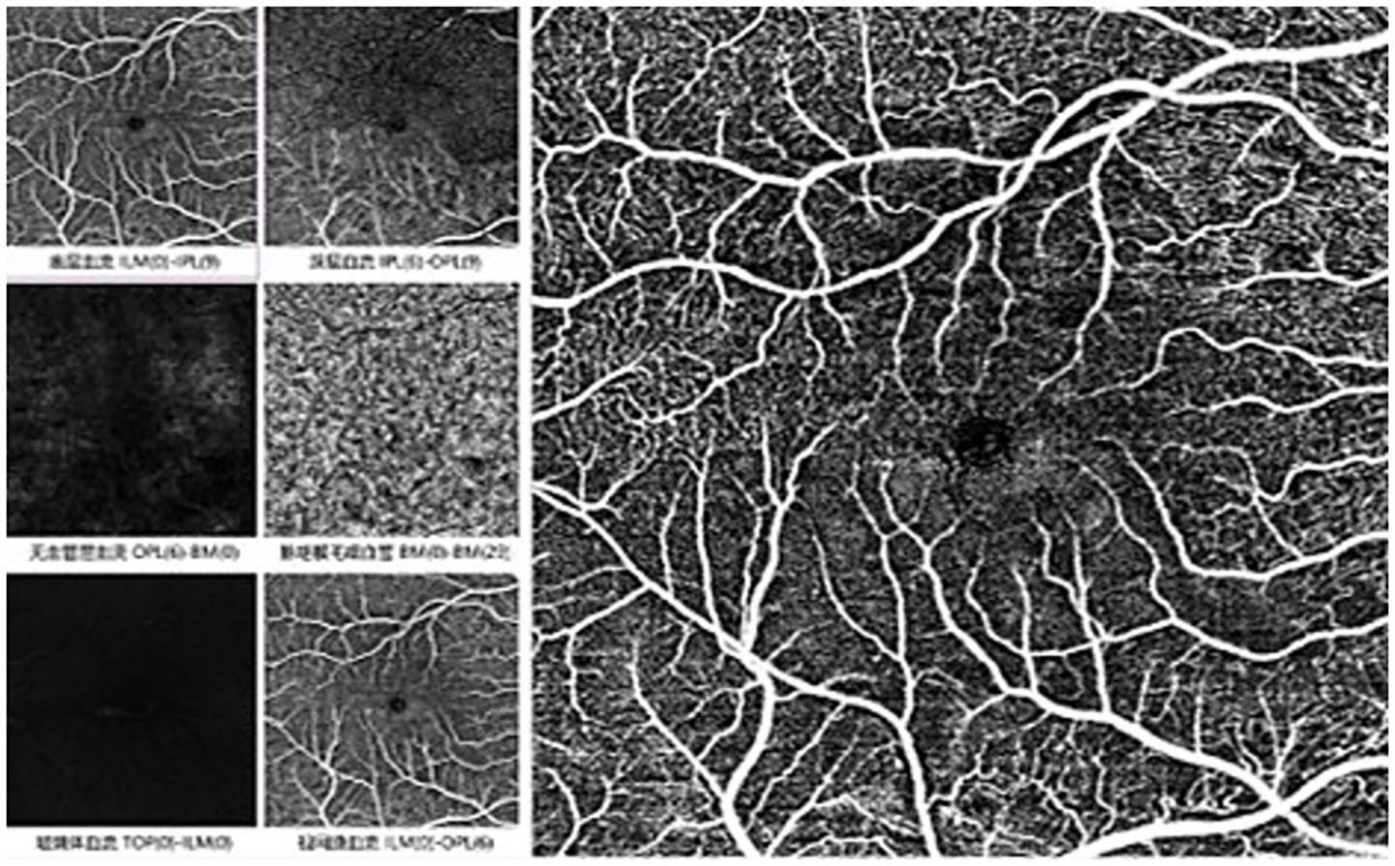
Pre-treatment macular findings in BRAO eye in OCTA: no perfusion zone observed in the superficial macular layer, with unclear arcades.

A 65-year-old Chinese man presented with a 5-day history of blurred vision and a black shadow in the upper visual field of the right eye, without associated ocular pain or redness. He had a 10-year history of hypertension but no history of diabetes. Two weeks prior, he had undergone phacoemulsification with intraocular lens implantation in the right eye. On initial examination, his uncorrected visual acuity was 0.6 (decimal) in both eyes, with best-corrected visual acuity (BCVA) of 1.0 in the right eye and no improvement in the left. Intraocular pressure was 10 mmHg in the right eye and 11 mmHg in the left. Fundus examination revealed cotton-wool spots in the posterior pole, macular edema, and retinal pallor in the inferior quadrant of the right eye (Figure 1a). Optical coherence tomography (OCT) of the macula showed increased inner retinal reflectivity and thickening with poorly defined retinal layers (Figure 1b). The left eye showed yellowish exudation nasal to the optic disc, vascular tortuosity, thinning, and arteriovenous crossing changes (Figure 1c). OCT of the left macula revealed no significant retinal edema (Figure 1d). OCT angiography (OCTA) of the right eye demonstrated non-perfusion in the superficial macular capillary plexus and disruption of the perifoveal vascular arcade (Figure 2).

**Figure 3:**
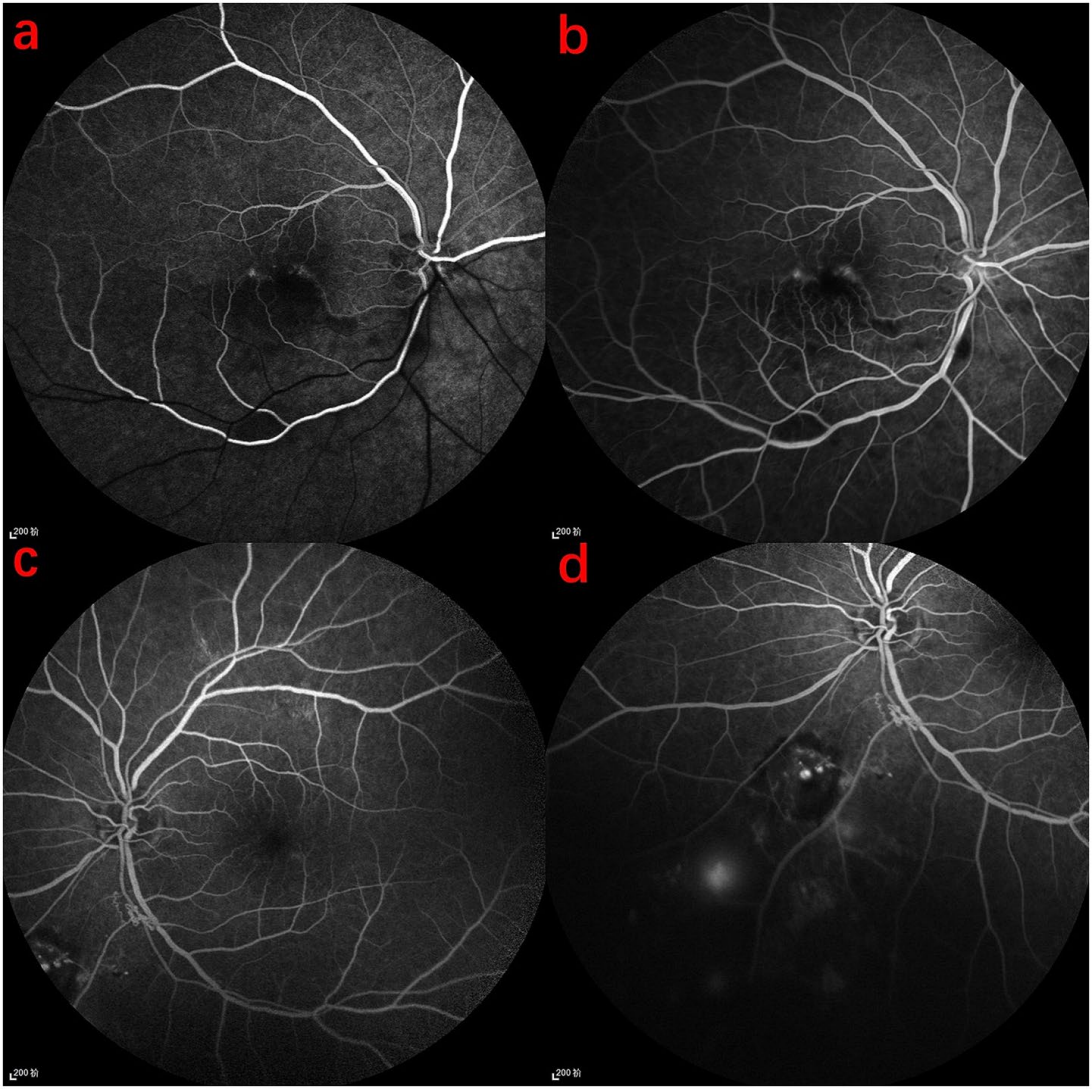
FFA findings in the patient. **(a)** Arterial phase of the right eye: normal arterial filling in the upper half, delayed filling in the lower half, with decreased fluorescence in the lower retinal background. **(b)** Late-phase macular area of the right eye: disruption of the retinal arcade and mild fluorescence accumulation. **(c)** Venous phase of the left eye: poorly defined refractive media, with localized subretinal hemorrhage inferior to the optic disc, showing patchy low fluorescence. **(d)** Evidence of microaneurysm leakage and neovascular leakage.

**Figure 4:**
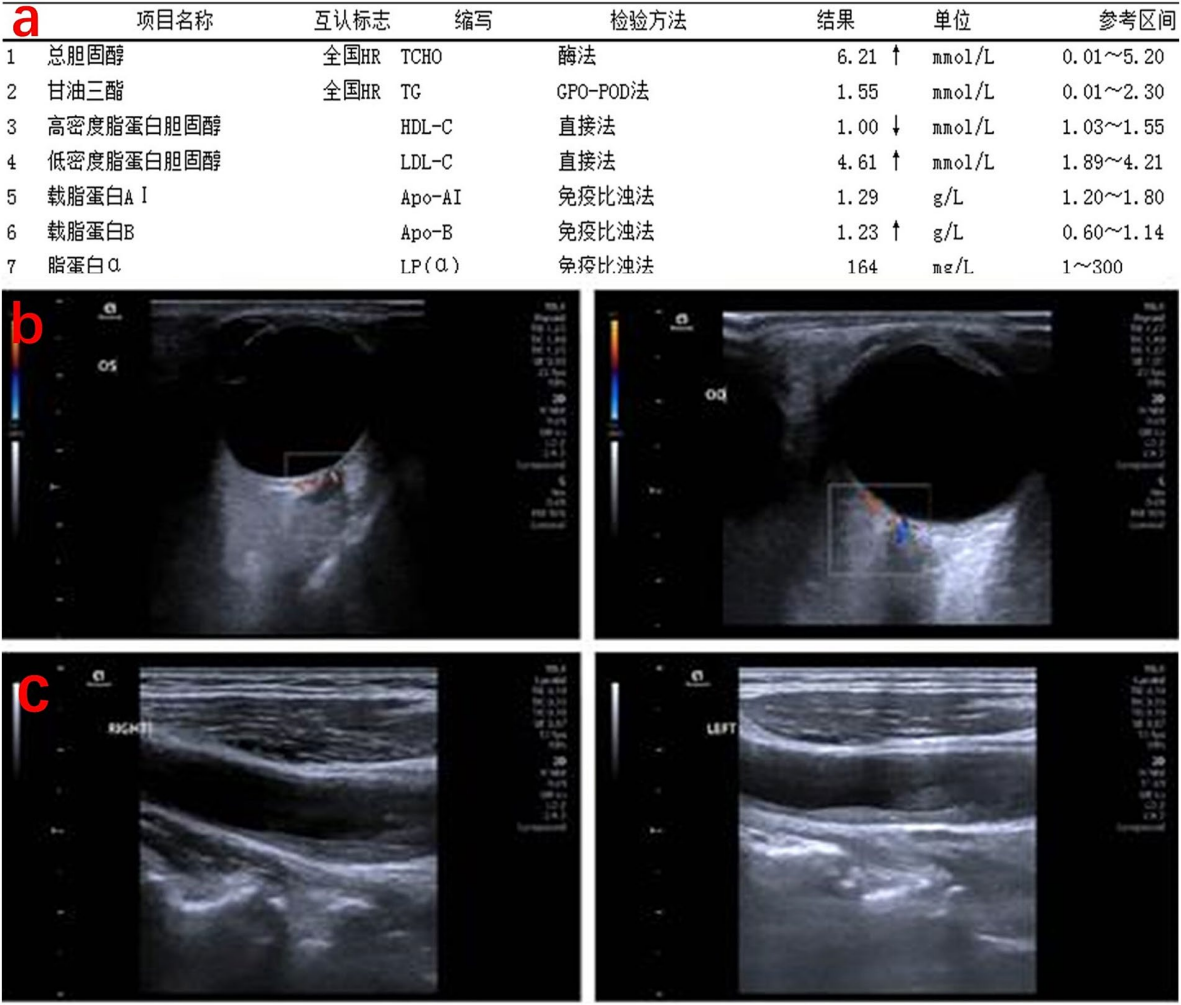
Lipid profile and vascular status of the patient. **(a)** Lipid profile: total cholesterol significantly elevated. **(b)** Posterior ocular vessel ultrasound: the right retinal central artery shows significantly reduced flow and velocity, with occlusion not ruled out. Reduced flow velocity in the right nasal ciliary posterior short artery. **(c)** Carotid ultrasound: bilateral carotid artery walls are irregular, with hypoechoic, hyperechoic, and mixed echogenic plaques visible in the common carotid artery, bifurcation, and the origin of the internal carotid artery. Blood flow filling defects are observed at the plaque sites.

Fluorescein angiography (FFA) revealed normal arterial filling in the superior retina and delayed arterial filling in the inferior half of the right eye, with corresponding hypofluorescence in the inferior background (Figure 3a). In the late phase, macular capillary dropout and mild fluorescein pooling were observed (Figure 3b). The left eye exhibited media opacity and, during the venous phase, patchy subretinal hemorrhage in the inferonasal quadrant with corresponding hypofluorescence (Figure 3c), along with subtle microaneurysm leakage and neovascular leakage (Figure 3d). Based on these findings, FFA confirmed branch retinal artery occlusion (BRAO) in the right eye and branch retinal vein occlusion (BRVO) in the left eye. Laboratory tests revealed significantly elevated total cholesterol (Figure 4a). Color Doppler ultrasound indicated reduced flow in the right posterior ciliary artery (Figure 4b), and carotid ultrasound showed bilateral atherosclerosis with plaque formation (Figure 4c). The patient received posterior sub-Tenon injections of dexamethasone (2.5 mg/0.5 mL) and anisodamine (5 mg/1 mL) once daily for five consecutive days. Meantime, this patient received superficial temporal artery injections of compound anisodine (2 mL per day) administered once daily for 7 days. Intravenous vinpocetine (20 mg diluted in 500 mL of 0.9% normal saline) was given via slow infusion once daily for 7 days to promote vasodilation. After treatment, fundus photography of the right eye showed improvement in cotton-wool spots, and OCT demonstrated a reduction in macular edema with improved morphology, though the retinal layers remained poorly defined (Supplementary Figures 1a,b). The left eye underwent retinal laser photocoagulation for ischemic BRVO, followed later by cataract surgery. Fundus photography showed laser scars surrounding a ring-shaped exudative lesion, with hemorrhages along the major vessels below the optic disc and vessel narrowing with peripheral hemorrhages in the inferonasal mid-periphery (Supplementary Figure 1c). OCT of the left macula remained unremarkable (Supplementary Figure 1d). The right eye was managed with vasodilators and medications to relieve vascular spasm and reduce intraocular pressure (not anti-glaucoma therapy). Upon discharge, the patient’s BCVA improved to 1.0 in the left eye and 0.8 in the right eye. He was advised to continue antihypertensive treatment and return for regular follow-up.

## Discussion

Retinal vascular occlusive disorders are among the most common retinal vascular diseases, second only to diabetic retinopathy (1). Among them, BRVO and BRAO are the most frequently encountered subtypes. While each of these conditions is well-documented individually, the occurrence of BRVO in one eye and BRAO in the fellow eye is exceedingly rare, with only a handful of cases reported in the literature. Such presentations underscore the possibility that both conditions may represent distinct manifestations of underlying systemic vascular pathology affecting the ocular circulation in different ways.

BRVO has a global prevalence of approximately 0.5 to 1.2%, predominantly affecting individuals over 60 years of age and most commonly involving the superotemporal quadrant of the retina (9). The pathogenesis of BRVO typically involves arteriovenous crossings where arteriosclerotic changes in the artery lead to compression of the adjacent vein, resulting in turbulent blood flow, endothelial injury, thrombosis, and eventual venous occlusion. Major risk factors include systemic hypertension, hyperlipidemia, diabetes mellitus, glaucoma, and smoking (3). In contrast, BRAO is most often caused by embolic phenomena originating from carotid atherosclerotic plaques or cardiac sources such as atrial fibrillation or valvular disease (10, 11). Some cases may also involve hypercoagulable states or inflammatory vasculitis, such as giant cell arteritis. BRAO typically presents with sudden, painless monocular vision loss, and fundus examination reveals localized retinal pallor and narrowed arteries, sometimes accompanied by the classic “cherry-red spot” if the fovea is involved (12).

A comprehensive literature search was conducted using PubMed^1^ to identify relevant studies published up to March 2025. The search strategy included combinations of the keywords “Branch Retinal Vein Occlusion,” “BRVO,” “Branch Retinal Artery Occlusion,” “BRAO,” “retinal vascular occlusion,” “retinal ischemia,” and “retinal vascular disorder.” Case reports, case series, and original studies were included to provide a broad overview of the clinical presentations, risk factors, and management strategies associated with the simultaneous or sequential occurrence of BRVO and BRAO in different eyes. Only articles published in English were considered for inclusion.

Various combinations of retinal arterial and venous occlusions due to different etiologies have been reported in the literature (13). These include mixed types such as central retinal vein occlusion (CRVO) with central retinal artery occlusion (CRAO), CRVO with cilioretinal artery occlusion, CRVO with BRAO, and BRVO with BRAO. In cases of CRVO/CRAO and CRVO/cilioretinal artery occlusion, when the site of occlusion is located within the optic nerve head, the mechanism of vascular compromise may be attributed to the anatomical relationship between the arteries and veins within the optic nerve. The lamina cribrosa serves as a confined anatomical space through which both the central retinal artery and vein enter the eye. Vascular congestion caused by thrombotic or embolic occlusion within this compartment may result in secondary compression of adjacent vessels, thereby leading to combined occlusive events. A case series by D. Schmidt supports this hypothesis, wherein 14 patients with acute unilateral vision loss due to combined retinal artery and vein occlusion were analyzed. In these cases, no emboli were detected, even though embolic events are generally the most common cause of isolated retinal artery occlusion. However, all reported combined occlusions occurred in the same eye; to date, there have been no documented cases of branch retinal vein occlusion and branch retinal artery occlusion occurring independently in opposite eyes.

Interestingly, most such patients share a history of chronic hypertension, a notable risk factor in the present case (3). Hypertension can lead to structural and functional changes in small arteries, predisposing patients to both venous compression (as in BRVO) and embolic events (as in BRAO). Furthermore, the temporal relationship between recent ocular surgery and the onset of BRAO in our patient raises the question of whether intraoperative fluctuations in intraocular pressure or postoperative inflammation may contribute to altered ocular hemodynamics, thereby triggering vascular occlusion. Although the evidence is limited, several reports suggest an increased risk of retinal vascular events in the perioperative period following intraocular surgeries such as cataract extraction (14, 15).

Treatment strategies differ for the two conditions. While effective therapies for BRAO remain elusive, early intervention such as ocular massage, anterior chamber paracentesis, and vasodilators may offer limited benefits. Previous case involving a 63-year-old male presented with sudden vision loss, whose condition improved solely with systemic anticoagulation (without ocular therapy) despite an elevated thrombotic risk, alongside an analysis of clinical findings using multimodal imaging and a review of similar literature (16). Antiplatelet therapy and systemic vascular risk management remain essential. For BRVO, evidence-based treatments include retinal laser photocoagulation, intravitreal injections of anti-VEGF agents or corticosteroids, and, in select cases, vitrectomy. For RVO eyes unresponsive to anti-VEGF therapy, the intravitreal dexamethasone implant represents a suitable alternative treatment (17). In our case, a combination of posterior sub-Tenon dexamethasone injection, compound anisodine, intravenous vinpocetine, and laser photocoagulation for the left eye resulted in measurable visual improvement in both eyes.

An important aspect of this case is the use of less commonly reported treatment modalities, including temporal artery injection of compound anisodine and intravenous vinpocetine. Compound anisodine is an anticholinergic alkaloid with *α*-receptor blocking properties, which has been used primarily in China to alleviate retinal vasospasm, improve ocular microcirculation, and stabilize the blood-retinal barrier. Temporal artery injection targets the perivascular autonomic nerve supply, potentially reducing local vasospasm and ischemia. Vinpocetine, a synthetic derivative of vincamine, is known for its vasodilatory, neuroprotective, and anti-inflammatory properties, and has been explored as a supportive therapy in ischemic cerebrovascular and retinal conditions. Though these therapies are not widely adopted in Western practice, several studies in Chinese ophthalmologic literature have reported improvements in visual function and retinal perfusion following such interventions (18–21). In our experience, this combined regimen contributed to rapid improvement in macular edema and visual acuity, with no observed adverse effects. Given the limited global exposure to these treatment methods, further prospective studies are warranted to validate their efficacy and safety in broader populations.

Although long-term outcome data for such bilateral but asymmetric occlusion cases are limited, BRAO generally has a poorer prognosis than BRVO, especially when the macula is involved or treatment is delayed. Continued follow-up with fundus examinations and imaging is crucial to detect possible complications such as neovascularization. Most importantly, the presence of dual ocular vascular occlusions should prompt comprehensive systemic evaluation and aggressive management of vascular risk factors to prevent future events such as stroke or myocardial infarction.

In summary, the coexistence of BRVO in one eye and BRAO in the other, although rare, serves as a clinical indicator of significant systemic vascular disease. A multidisciplinary approach involving ophthalmologists, neurologists, and cardiologists is warranted to manage these patients holistically. Early diagnosis and tailored intervention may not only preserve vision but also reduce systemic morbidity and mortality associated with underlying vascular pathology.

## Data Availability

The original contributions presented in the study are included in the article/Supplementary material, further inquiries can be directed to the corresponding authors.

## Footnotes

^1^ https://pubmed.ncbi.nlm.nih.gov/

## Supplementary material

SUPPLEMENTARY FIGURE 1 Follow-up examination at discharge. **(a)** Fundus photography: cotton-wool spots in the BRAO eye have improved compared to previous images. **(b)** OCT scan: macular edema in the BRAO eye has decreased, with improved morphology, though the layers remain unclear. **(c)** Fundus photography: in the BRVO eye, the circular exudative lesion is surrounded by laser treatment, with hemorrhage around large vessels below the optic disc and signs of vessel narrowing and hemorrhage in the nasal mid-periphery. **(d)** OCT scan: no significant morphological abnormalities observed in the BRVO eye.
