# Supplementary figures and images for: Unilateral branch retinal vein occlusion and contralateral branch retinal artery occlusion: a case report and comprehensive literature review
Source: Front Med (Lausanne). 2025 Jul 22;12:1615871. doi: 10.3389/fmed.2025.1615871 (PMC12321791; doi:10.3389/fmed.2025.1615871)

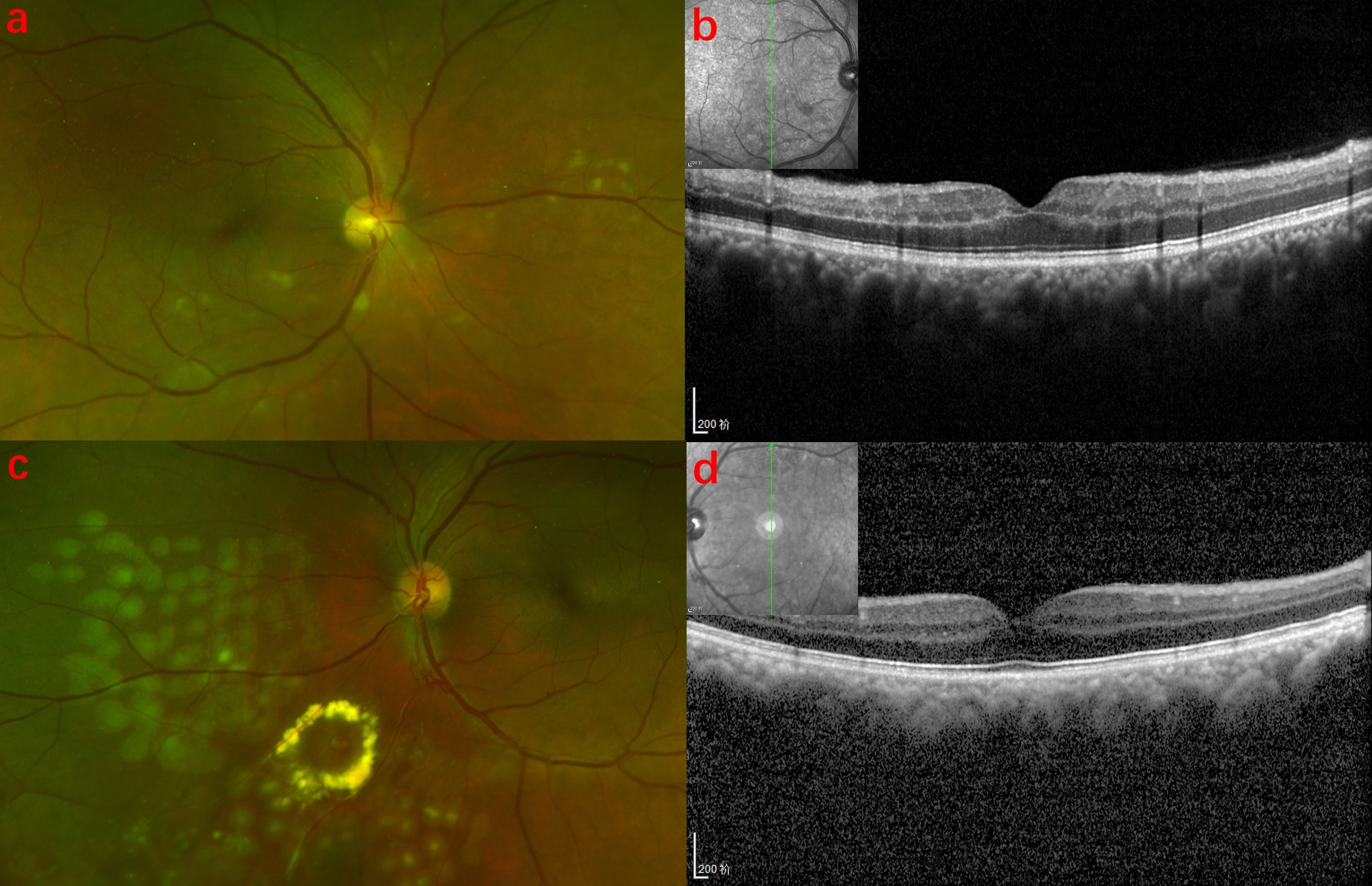

Supplement: SUPPLEMENTARY FIGURE 1 — Follow-up examination at discharge. (a) Fundus photography: cotton-wool spots in the BRAO eye have improved compared to previous images. (b) OCT scan: macular edema in the BRAO eye has decreased, with improved morphology, though the layers remain unclear. (c) Fundus photography: in the BRVO eye, the circular exudative lesion is surrounded by laser treatment, with hemorrhage around large vessels below the optic disc and signs of vessel narrowing and hemorrhage in the nasal mid-periphery. (d) OCT scan: no significant morphological abnormalities observed in the BRVO eye. [file Image_1.jpeg]
